# A systematic comparison of human mitochondrial genome assembly tools

**DOI:** 10.1186/s12859-023-05445-3

**Published:** 2023-09-13

**Authors:** Nirmal Singh Mahar, Rohit Satyam, Durai Sundar, Ishaan Gupta

**Affiliations:** 1 Department of Biochemical Engineering and Biotechnology, Indian Institute of Technology, New Delhi, 110016 India; 2 Jamia Millia Islamia, Jamia Nagar, Okhla, New Delhi, 110025 India

**Keywords:** Mitochondria, Genome, Benchmark, Assembly

## Abstract

**Background:**

Mitochondria are the cell organelles that produce most of the chemical energy required to power the cell's biochemical reactions. Despite being a part of a eukaryotic host cell, the mitochondria contain a separate genome whose origin is linked with the endosymbiosis of a prokaryotic cell by the host cell and encode independent genomic information throughout their genomes. Mitochondrial genomes accommodate essential genes and are regularly utilized in biotechnology and phylogenetics. Various assemblers capable of generating complete mitochondrial genomes are being continuously developed. These tools often use whole-genome sequencing data as an input containing reads from the mitochondrial genome. Till now, no published work has explored the systematic comparison of all the available tools for assembling human mitochondrial genomes using short-read sequencing data. This evaluation is required to identify the best tool that can be well-optimized for small-scale projects or even national-level research.

**Results:**

In this study, we have tested the mitochondrial genome assemblers for both simulated datasets and whole genome sequencing (WGS) datasets of humans. For the highest computational setting of 16 computational threads with the simulated dataset having 1000X read depth, MitoFlex took the least execution time of 69 s, and IOGA took the longest execution time of 1278 s. NOVOPlasty utilized the least computational memory of approximately 0.098 GB for the same setting, whereas IOGA utilized the highest computational memory of 11.858 GB. In the case of WGS datasets for humans, GetOrganelle and MitoFlex performed the best in capturing the SNPs information with a mean F1-score of 0.919 at the sequencing depth of 10X. MToolBox and NOVOPlasty performed consistently across all sequencing depths with a mean F1 score of 0.897 and 0.890, respectively.

**Conclusions:**

Based on the overall performance metrics and consistency in assembly quality for all sequencing data, MToolBox performed the best. However, NOVOPlasty was the second fastest tool in execution time despite being single-threaded, and it utilized the least computational resources among all the assemblers when tested on simulated datasets. Therefore, NOVOPlasty may be more practical when there is a significant sample size and a lack of computational resources. Besides, as long-read sequencing gains popularity, mitochondrial genome assemblers must be developed to use long-read sequencing data.

**Supplementary Information:**

The online version contains supplementary material available at 10.1186/s12859-023-05445-3.

## Background

### General introduction

Mitochondrial DNA (mtDNA) is present in all aerobic eukaryotes 1, with human mtDNA's size being ~ 16 kbp, encoding for 13 proteins 2. The first draft of human mitochondrial genome assembly was published by Anderson et al. in 1981 2, leading to advances in several fields such as forensics 3, pharmaceuticals 4, anthropology 5 and evolution 6. However, this reference mitochondrial genome, often called Cambridge Reference Sequence (CRS), is a contemporary European sequence that has not been revised since 1999 7. After the availability of the complete Neanderthal mitochondrial genome 8, Behar et al. proposed the usage of the Reconstructed Sapiens Reference Sequence (RSRS) over the revised Cambridge Reference Sequence (rCRS). RSRS was constructed using ~ 8 K human mitochondrial genomes 9; however, this preposition was later refuted by Bandelt et al. 10

The deluge of sequencing data in the past decade enables us to study more complex attributes of the mitochondrial genome at the population level. Heteroplasmy, the co-occurrence of multiple mitochondrial DNA haplotypes within an individual 11, was previously considered rare. However, with the advent of high throughput sequencing, we can now enumerate the extent of polymorphism of the mitochondrial genome at the population level 12. Technological advances in mtDNA sequencing, such as isothermal rolling circle amplification 13 and targeted nanopore sequencing 14_,_ now enable selective sequencing of the mitochondrial genome.

The task of assembling organelle genomes is critical. The mitochondrial genome has been extensively utilized in examining non-model species evolution 15, studying phylogenetics 16 and species identification research 17. Assembly of the circular mitochondrial genome has also been shown to help in understanding the evolution of frogs 18.

Since mitochondrial reads represent a significant proportion of reads (up to 25%) in Next-Generation whole genome sequencing data, they can be considered one of the most extensively sequenced genomes in eukaryotes 19. In human specific context, they can be segregated from the sequencing data and leveraged to build mitochondrial Pan genomes 20, study heteroplasmy 1, 12, and the hypermutation process 21. Human mitochondrial genome sequences are also used to analyze human lineages and migration trends 22, 23, study population genetics 24, research human mitochondrial illnesses 25, and conduct forensic science research 26. It is a mainstay in forensic research as some locations of mtDNA evolve 5–10 times faster than nuclear genes, and these regions are routinely typed in forensic studies 27. Furthermore, having access to multiple mitogenomes allows us to conduct large-scale comparative research 19.

Mitochondrial genomes have been assembled using various methods, and in this study, we specifically focus on assembling human mitochondrial genomes. Typically, two main approaches are involved in deriving a mitochondrial genome from whole-genome sequencing (WGS) data: The first approach is reference genome mapping to extract mitochondrial reads from the sequencing data, followed by assembly and resolution of the specific circular structure 28, 29. This can be achieved by mapping the reads to the current reference mitochondrial genome (rCRS). The reference-based category has the benefit of requiring less memory and running time. The second strategy, also known as de novo assembly, uses increased coverage of reads coming from the mitochondria and therefore does not rely on the existence of a reference genome 30. The most common reads may be extracted using a k-mer analysis. One of the mitochondrial genome assembling tools utilizing the k-mer-based approach is MitoZ 31. These approaches have the benefit of being widely applicable as they can be utilized in assembling genomes of novel species. However, nuclear-embedded mitochondrial DNA sequences (NUMTs) 32, the mitochondrial DNA sequences integrated into the nuclear genome, present a significant challenge in the de novo assembly. The inherent sequence similarity between NUMTs and mitochondrial DNA can cause misassignments of reads during the assembly process, leading to excessive runtimes and a higher likelihood of generating fragmented mitochondrial assemblies. A hybrid strategy, for instance, utilized by NOVOPlasty 33, combines the two approaches mentioned above by concurrently assembling the reads based on k-mers and utilizing a mitochondrial reference gene as a seed. The benefit of the seed-and-extend category is that it can be used for any known species; all that is required, in addition to the paired-end reads in FASTQ format, is a brief seed sequence of that species or any closely related species. However, because of the read length limitation offered by short-read sequencing, it struggles to decode repetitive regions seen in some mitochondrial genomes 34.

### Purpose and scope of this study

Though several tools were developed for mitogenome assembly, this study, for the first time, evaluates the performance and efficacy of open-source command-line tools currently available for performing de novo and reference-based assembly from raw genomic data for human mitochondrial genomes. Benchmarking online servers for human mitochondrial assembly is beyond the scope of the present study. Further, we limited our benchmark to readily configurable tools, straightforward to install and actively maintained. We restrict the input to paired-end Illumina data as contemporary sequencing technologies often produce them.

We tested all the tools on six publicly available human datasets with various down-sampling rates and simulated datasets. In this study, we have observed that the performance of the majority of tools was enhanced considerably by downsampling the sequencing data to an average sequencing depth of 10X. We also believe that the performance of some tools can be improved by fine-tuning their parameters. However, this was beyond the scope of this study.

## Methodology

### Data availability

Docker images of all the tools used in this study have either been present or deposited on Dockerhub 35, and all the tools are hosted on GitHub (Table 1).

**Table 1:** Information about the mitochondrial genome assemblers utilized in this study

Tool	Source repository	Links for docker images available online	Reference genome dependency	Main approach	Programming language	License	Miscellaneous features	Multithreading support	Input data
ARC	https://github.com/ibest/ARC.git	https://hub.docker.com/repository/docker/nirmal2310/arc_docker/	Yes	Mapping to the reference genome followed by *de novo* assembly.	Python 2	Apache License 2.0	NA	Yes	Paired-End Illumina Fastq Files (Unzipped)
GetOrganelle	https://github.com/Kinggerm/GetOrganelle.git	https://hub.docker.com/repository/docker/nirmal2310/getorganelle_docker/	Yes	Mapping to the reference genome followed by *de novo *assembly.	Python 3	GNU GPL v3.0	NA	Yes	Paired-End/Single-End Illumina Fastq Files (Can be Gzipped)
IOGA	https://github.com/holmrenser/IOGA.git	https://hub.docker.com/repository/docker/nirmal2310/ioga_docker/	Yes	Mapping to the reference genome followed by *de novo *assembly.	Python 2	GNU Affero GPL v3.0	NA	Yes	Paired-End/Single-End Illumina Fastq Files (Can be Gzipped)
MEANGS	https://github.com/YanCCscu/MEANGS.git	https://hub.docker.com/repository/docker/nirmal2310/meangs_docker/	No	*De novo *Assembly	Python 3	GNU GPL v3.0	NA	Yes	Paired-End/Single-End Illumina Fastq Files (Can be Gzipped)
MITObim	https://github.com/chrishah/MITObim.git	https://hub.docker.com/repository/docker/nirmal2310/mitobim_docker/	Yes	Mapping to the reference genome followed by *de novo *assembly.	Perl	MIT LICENSE	NA	No	Paired-End/Single-End Illumina Fastq Files (Paired-End should be Interleaved and Unzipped)
MitoFlex	https://github.com/Prunoideae/MitoFlex.git	https://hub.docker.com/repository/docker/nirmal2310/mitoflex_docker/	No	*De novo *assembly	Python 3	GNU GPL v3.0	Genome annotation	No	Paired-End/Single-End Illumina Fastq Files (Can be Gzipped)
MitoZ	https://github.com/linzhi2013/MitoZ.git	https://hub.docker.com/repository/docker/nirmal2310/mitoz_docker/	No	*De novo *assembly	Python 3	GNU GPL v3.0	Genome annotation	Yes	Paired-End/Single-End Illumina Fastq Files (Can be Gzipped)
MToolBox	https://github.com/mitoNGS/MToolBox.git	NA	Yes	Mapping to the reference genome followed by *de novo *assembly.	Python 2	GNU GPL v3.0	Haplogroup Prediction, Variant Calling	Yes	Paired-End/Single-End Illumina Fastq Files (Can be Gzipped)
NOVOPlasty	https://github.com/ndierckx/NOVOPlasty.git	https://hub.docker.com/repository/docker/nirmal2310/novoplasty_docker/	Yes	Seed and extend approach	Perl	NA	NA	No	Paired-End/Single-End Illumina Fastq Files (Can be Gzipped)
			(Can be a single gene sequence)						
ORG.Asm	https://git.metabarcoding.org/org-asm/org-asm.git	https://hub.docker.com/repository/docker/nirmal2310/org-asm_docker/	Yes	Seed and extent approach	Python 3	CeCILL LICENSE	NA	No	Paired-End/Single-End Illumina Fastq Files (Can be Gzipped)
			(Can be a single gene sequence)						

Detailed information about the mitochondrial assemblers used in this study has been given in Table 1. The Docker container for MToolBox is unavailable via Docker Hub but can be built locally using the Docker file provided in the GitHub repository: https://github.com/Nirmal2310/Mitochondrial_Benchmarking_study. The information about GitHub commits for the tools has been provided in Additional file 2: Table S6

Simulated data have been used to collect the run metrics for various run parameters, and the guidelines for computational benchmarking have been followed while conducting this study 36.

### Tool selection

In this study, we have only included those tools for assembling a mitochondrial genome that uses Short Paired End Reads. Tools must be available as open source and must allow command-line execution. Only tools that offered command line interface (CLI) were open-source and were actively maintained were included. Tools having graphical user interfaces weren't included in the study since they couldn't be used to automate the assembly of tens of thousands of samples. The tools that met all criteria mentioned above are listed below:

ARC v1.1.4-beta 37, Get-Organelle v1.7.5.1 38, MEANGS v1.0.1 39, IOGA 40, MITObim v1.9.1 41, MitoZ v2.3 31, NOVOPlasty v4.3.1 33, MToolBox v1.2 29, MitoFlex v0.2.9 42 and ORG.Asm v2.2 43.

These assemblers have been successfully used in assembling organelle genomes of various species [44–46; however, we are focusing on the performance of these assemblers for assembling human mitochondrial genomes.

Some CLI-based tools that couldn't be part of this study include Organelle PBA 47, Norgal 30 and mitoMaker 48. Organelle PBA is designed to deal with long reads sequencing data generated by PacBio technology. Norgal and mitoMaker failed initial testing of assembling mitochondrial genomes and hence were excluded from the study. Similarly, web-based GUI-based SMART 49 software is also available but doesn't fulfil this study's scope.

### Our setup

We used default parameter settings to compare all of the assembly tools equitably. The input sequencing data included a pair of FASTQ files, one representing forward (R1.fastq) and the other representing reverse (R2.fastq) read. The output files generated were named after the tool that produced them. Additionally, we created separate conda 50 environments for each tool. Finally, to get the run metrics for each run (CPU usage, memory usage etc.), we generated docker images for each tool based on Ubuntu 18.04 base image preloaded with all the dependencies and software. The benchmark was performed on an AMD EPYC 7502 processor with 32 cores and 512 GB RAM.

### Data

#### Simulated data

We simulated reads based on the human mitochondrial genome retrieved from the recently published complete human genome 51 (GenBank accession number CP068254.1) to avoid errors produced by sequencing runs and biological variation. To generate these perfect reads, we used InSilicoSeq NGS simulator 52 with the Novaseq error model (150 bp paired) and various coverage models offered by the simulator using the *–coverage* parameter. Previous studies suggest that 53 whole genome sequencing results in a mean read depth between 1200 and 4000X for the mitochondrial genome. The high coverage for mitochondrial reads is due to a cell's high copy numbers of the mitochondrial genome. Hence, we generated simulated data containing 115 K, 175 K and 225 K numbers of reads to get the mean depth of 2000X, 3000X, 4000X respectively.

#### Real data

We selected six whole genome sequencing datasets (NA12877, NA12878, NA12889, NA12890, NA12891, NA12892) from the study "Whole genome sequencing and variant calls from Coriell CEPH/UTAH 1463 family to create a platinum standard comprehensive set for variant calling improvement" 54, sequenced by Illumina Cambridge Ltd. with the sequencing depth varying from 46 to 55X. We down-sampled the six paired-end FASTQ files for further analysis to a mean sequencing depth of 10X, 20X, 30X and 40X, respectively.

### Evaluation criteria

#### Computational resources

We recorded each assembler's peak CPU and memory load and the size of the assembly files. All assembly tools were run on our docker image configuration using 2, 4, 8, and 16 threads for various simulated data sets (115 K, 175 K, and 225 K reads).

Besides, we observed that several tools used more threads than were specified during the initial run, so we used the *–cpu* argument of the *docker run* command to reduce the overhead. We estimated each configuration's memory consumption and CPU usage using the docker stats command, which produces a live stream of a container's runtime metrics.

### Qualitative

The qualitative assessment of all the tools used in this study was based on the Journal of Open-Source Software (JOSS) 55 reviewer guidelines. The evaluation was done based on the following questions:

1. Are the tools easy to install?
2. Is there proper documentation for running the tool or a test dataset to check the installation?
3. Is the tool well maintained (issues answered, continuous update)?
4. Is the tool Open Source?

These questions were answered "good", "bad", and "okay" based on the experience while installing and running the tool. If the tool is available as a CONDA package, bundled into a container, or has pre-compiled binaries, that tool will be considered "good". An "okay" installation tag would refer to a scenario where a custom script is available to download and compile all the dependencies needed for the tool. However, a tool is tagged "bad" when the tool is unable to install using improper and insufficient documentation and requires intensive debugging and dependencies resolution. Detailed information about the criteria for these evaluations has been explained in Additional file 1.

### Quantitative

For assessing the quality of the genomes assembled by each assembler, we used the scoring scheme used by Freudenthal et al*.*^44^ . In this study, the authors compared assembling tools for chloroplast genomes. Since mitochondrial genomes are also extra-chromosomal, we found it appropriate to use the same method for mitochondrial genomes. This scoring scheme contains four metrics, each contributing one-fourth of the total score: completeness, correctness, repeat resolution and continuity.

1 $${\text{SCORE}} = \frac{1}{4}\left\{ {COV_{ref} + COV_{qry} + \min \left( {\frac{{COV_{qry} }}{{COV_{ref} }},\frac{{COV_{ref} }}{{COV_{qry} }}} \right) + \frac{1}{{N_{contigs} }}} \right\}$$

To estimate the completeness of the assembled mitochondrial genome, the coverage of the assembled mitochondrial genome with respect to the reference genome was calculated (COV_ref_). The assembled mitochondrial genome was aligned with the reference mitochondrial genome (GenBank accession number CP068254.1) using minimap2 v2.17 56, and the coverage was calculated using bedtools v2.30.0 genomecov module 57. This metric represents how many bases in the assembled genome are mapped to the reference genome. The second metric, correctness, was calculated by mapping reference to the assembled genome and calculating the coverage (COV_query_). Repeat resolution was calculated using {min (COV_query_/COV_ref_, COV_ref_/COV_query_)}, representing the difference between the length of the assembled genome and the reference genome. The number of contigs estimated the fourth metric continuity; the higher the number of contigs lower the continuity. We also applied this scoring schema on the assemblies generated using the down-sampled data to gauge if down-sampling is improving the performance of the assemblers or not. For downsampling the raw sequencing data, the *reformat.sh* module of bbtools (v. 37.62) 58 was used. The final assemblies were also compared with the reference mitochondrial genome using QUAST v5.0.2 59 since it is a well-known assessment tool for the assemblies. The perfect assemblies obtained by calculating the score using Eq. 1 were assessed for misassemblies, INDELs and mismatches, and the resulting metrics are stored in Additional file 2: Table S7.

We sought to determine whether the assembled mitochondrial genomes had any variations or were identical to the reference we provided since most of these assemblers are reference-based. Only Single Nucleotide Polymorphisms (SNPs) were probed for in the assembled genomes in this work, and they were compared with variants obtained from the raw sequencing data. The SNPs were called from raw sequencing data using Mutserve (v.2.0.0-rc13) 60, given its accuracy for mitochondrial variant calling as assessed in a benchmark study of mitochondrial variant calling tools 61. Mutserve utilizes the mapped bam file and calls SNPs while comparing the mapped reads with the reference mitochondrial genome. The SNPs from the assembled genomes were called using the *show-snps* utility of MUMmer (v.3.1) 62. The output of show-snps was converted to a VCF file using *all2vcf*^63^ . Lastly, we compared the SNPs from the assembled genomes with those from the raw sequencing data using bedtools intersect to get the True Positive, False Positive and False Negative metrics. These metrics were used to calculate the F1-score, the harmonic mean of precision and recall for each assembler to assess their variant retaining performance.

- True Positive (TP): SNPs common in both analyses.
- False Positive (FP): SNPs are only listed in the MUMmer output.
- False Negative (FN): SNPs are only listed in the Mutserve output.
  2 $$Precision =\frac{True \, Positives}{True \, Positives + False \, Positives}$$
  3 $$Recall = \frac{True \, Positives}{True \, Positive + False \, Negatives}$$
  4 $$F1 \, Score = 2*(\frac{precision*recall}{precision + recall})$$

## Results

### Performance metrics

All of the short-read assemblers examined in this study were compared regarding execution time, memory requirement, and CPU utilization.

### Time requirements

**Fig. 1:**
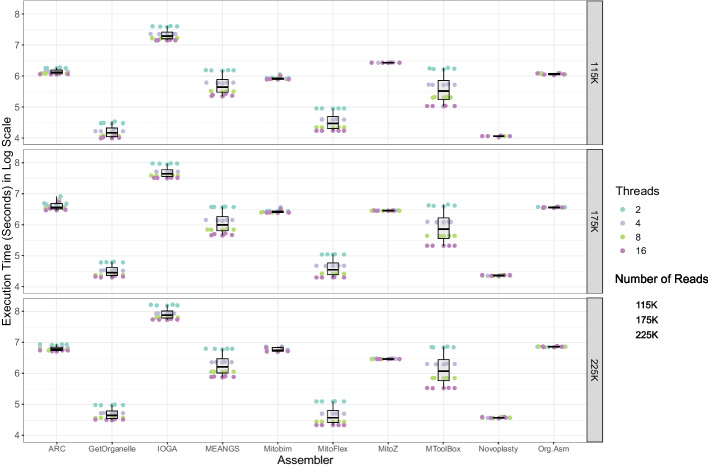
Computation time depending on the number of threads and size of input data. The box and swarm plots depict the differences in run time for various thread counts and input data sizes for the ten assemblers. MitoFlex took the least execution time, utilizing 16 computational threads, followed by GetOrganelle and NOVOPlasty. However, IOGA took the longest time to assemble mitochondrial genomes irrespective of read abundance or the number of threads specified

Significant differences in total execution time were observed for the same input data across the different tools (Fig. 1). Aside from tool differences, input data and the number of threads used significantly impacted the time required; the execution time ranged from 1.15 min for MitoFlex to 1.032 h for IOGA. For the highest computational setting of 16 computational threads with the simulated dataset of 4000X read depth, IOGA took an execution time of approximately 39 min, whereas MitoFlex took approximately 1.3 min. This trend of IOGA taking the longest execution time and MitoFlex taking the least execution time was consistent across all the other run settings. MitoFlex was followed by GetOrganelle and NOVOPlasty in terms of execution time. Not all tested tools benefited from having access to multiple computational threads. NOVOPlasty, ORG.Asm and MITObim do not support multithreading.

### Memory and CPU Usage

**Fig. 2:**
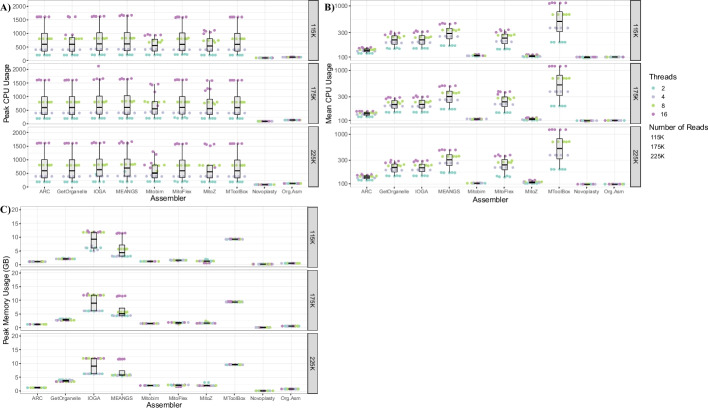
Performance metrics of all the mitochondrial assemblers with simulated data as the input data. **A** Box and Swarm Plots showing the peak CPU usage (1 Thread = 100%) for all the assemblers at various input data sizes and the number of threads. The plot shows variation in the peak CPU usage with the number of threads specified. **B** Box and Swarm plots of each assembler's average CPU usage. The plots clearly show a difference between mean and peak CPU usage, indicating that the assemblers do not use all of the threads provided by the user throughout the entire run. **C** Box and Swarm Plots showing the peak memory usage for all the assemblers at various input data sizes and the number of threads. An increase in the RAM requirement can be seen with the increment in the input data size. Additional file 2: Table S1 provides detailed information on the computational resources used by each tool for simulated datasets

Based on the same input data set and thread count, the peak CPU and memory usage and average CPU usage were recorded for all assemblers (Fig. 2). Most programs benefited from more threads for the input data size. However, the increment in the CPU threads increases the peak memory required in most cases. Again, for the same run setting of 16 computational threads and a simulated dataset of 4000X read depth, IOGA utilized the highest computational memory of 11.87 GB. In contrast, NOVOPlasty utilized the least computational memory of approximately 0.17 GB. This trend of IOGA utilizing the highest computational memory and NOVOPlasty utilizing the least computational memory was consistent across all the other run settings.

### Qualitative

**Fig. 3:**
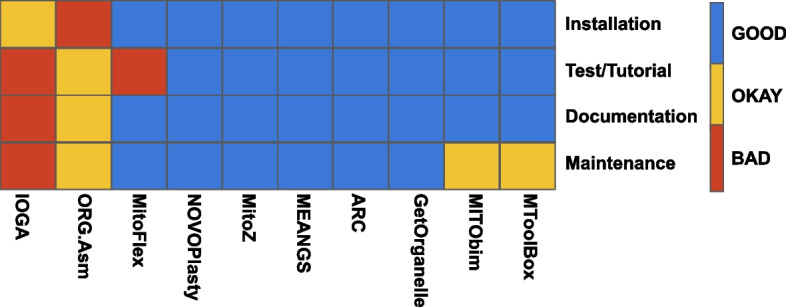
Overview of the results of the qualitative usability evaluation. Each tool was categorized as GOOD, BAD or OKAY based on specific metrics defining the ease of installation, test run, documentation and code maintenance﻿

On average, the user experience, in terms of installation and running of the analyses, was evaluated as "Good" for all the tools considered in this study. (Fig. 3).

### Quantitative

For a quantitative evaluation, we tested the capacity of all programs to assemble the human mitochondrial genome based on different input data. Input data were generated from the current reference mitochondrial genome or downloaded from sequencing repositories.

### Simulated data

**Fig. 4:**
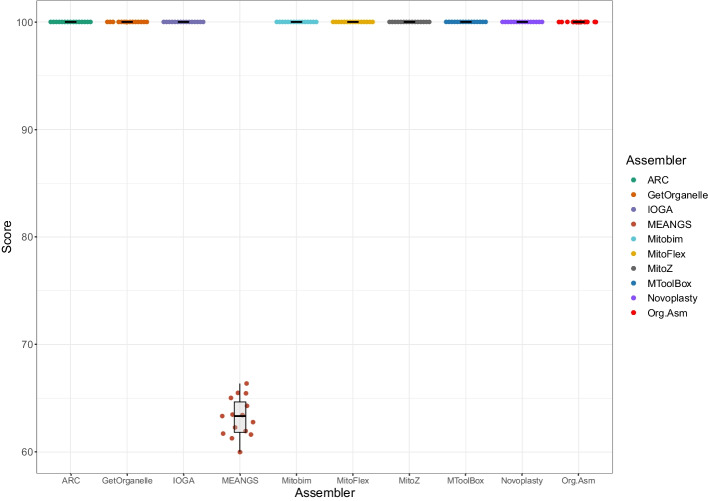
Score of the mitochondrial assemblies produced using simulated datasets. The Box and Swarm Plot describe the assemblies' scores produced by the tools. The score was calculated based on the mapping statistics of the assembly with the reference mitochondrial genome (Eq. 1). Except for MEANGS, all the other tools generated high-quality mitochondrial assemblies (score > 99). Scores for all the assemblies generated are given in Additional file 2 Table S2

The datasets with various sequencing depths were simulated using the mitochondrial genome retrieved from the latest complete human genome sequence (T2T-CH13). Assemblies obtained from the assemblers were compared with the reference mitochondrial genome, and a score was calculated based on Eq. 1. Except for MEANGS, all the other tools generated high-quality perfect assemblies (score $\ge$ 99) for every simulated dataset (Fig. 4).

### Publicly available datasets

**Fig. 5:**
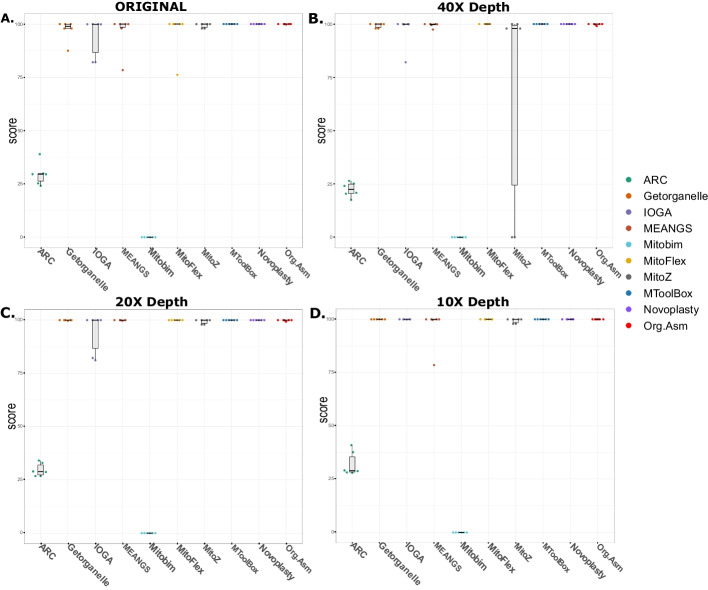
Comparing the effect of down sampling on the score of the mitochondrial assemblers. The bar and swarm plots in Figures A, B, C, and D show the scores of the assemblies generated by the assemblers using original data and down-sampled data of mean sequencing depth of 40X, 20X, and 10X, respectively. Most of the tool's performance increased for all six datasets at a sequencing depth of 10X

**Fig. 6:**
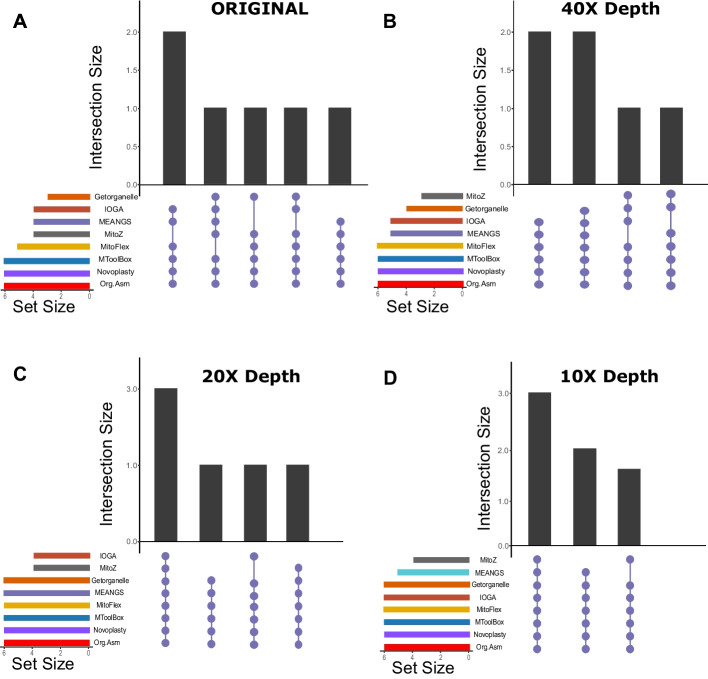
Comparison of effect of down sampling on the perfect assemblies generated by the assemblers. UpSet plot comparing perfect assemblies (score > 99) generated by different mitochondrial assemblers for original samples and down-sampled to the sequencing depths of 40X, 20X and 10X, respectively. Most tools produced high-quality assemblies for most datasets at a mean sequencing depth of 10X

The assemblies generated by the ten assemblers were scored based on their alignment with the reference mitochondrial genome. We observed significant variation in the performance of the tools considered in this study, among all the tools MToolBox, NOVOPlasty, and ORG.Asm assembled perfect genomes for all six samples (Fig. 5A). However, since some of the tools failed to assemble the genomes for all six samples, we examined the impact of downsampling on their performance. Most of the assemblers demonstrated higher quality and produced perfect assemblies at a sequencing depth of 10X, confirming that downsampling the data enhances the performance of the assemblers (Fig. 5). The UpSet plot, which compares the perfect assemblies generated by the assemblers, also illustrates the impact of downsampling. At a sequencing depth of 10X, most assemblers produced high-quality assemblies for all six samples (Fig. 6).

It is noteworthy that ARC and MITObim did not produce perfect assemblies at any sequencing depth (Table 2, Additional file 2: Table S4). The perfect assemblies were compared with the reference mitochondrial genome using QUAST v5.0.2 to assess the assemblies for misassemblies, INDELs and mismatches. Apart from the assemblies obtained from MEANGS, no other assemblies showed misassemblies compared to the reference mitochondrial genome. This information is available in Additional file 2: Table S7.

**Table 2:** Scores of the assemblies generated by each mitochondrial genome assembler for samples with a mean sequencing depth of 10X

Sr. no.	Assembler	Median	IQR	Perfect assemblies
1	ARC	28.85	7.20	0
2	GetOrganelle	99.99	0.01	6
3	IOGA	99.98	0.03	6
4	MEANGS	99.97	0.19	5
5	MITObim	0	0.00	0
6	MitoFlex	99.99	0.001	6
7	MitoZ	99.97	1.58	4
8	MToolBox	100.00	0.00	6
9	NOVOPlasty	99.99	0.01	6
10	ORG.Asm	99.99	0.03	6

Overall GetOrganelle, IOGA, MitoFlex, MToolBox, NOVOPlasty and ORG.Asm produced the perfect assemblies (score > 99) for all six datasets, followed by MEANGS and MitoZ. MITObim and ARC failed to produce good-quality assemblies for these datasets. Information about the scores obtained for the original, 40X and 20X sequencing data assemblies is available in the Additional file 2: Table S4

Additional file 2: Table S3 details the number of reads, mitochondrial genome sequence depth, and mean sequencing depth.

**Fig. 7:**
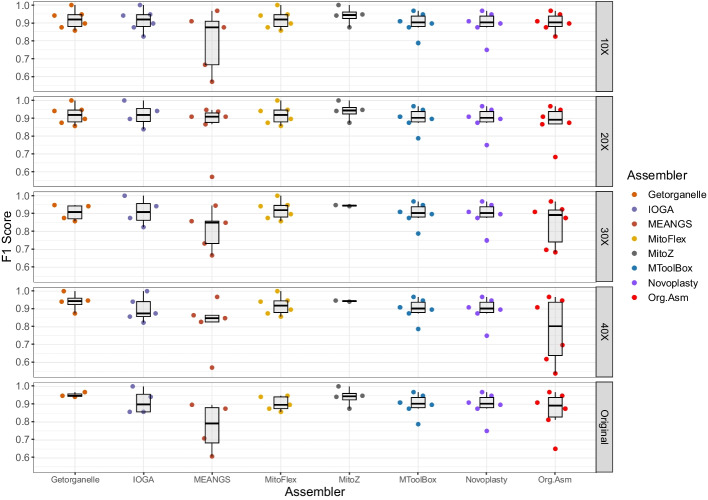
Comparison of F1-scores for SNPs calling using the assembled genomes and using the sequencing data. A box and swarm plot depicts F1-scores for precision and recall for SNPs calling using perfect assemblies (Score ≥ 99) produced by the ten mitochondrial assemblies methods using MUMmer and the original sequencing data using Mutserve. Overall, GetOrganelle and MitoFlex had the highest mean F1 scores (0.919) at the 10X sequencing depth, whereas MToolBox and NOVOplasty performed consistently at all sequencing depths with a mean F1 score of 0.897 and 0.890, respectively. Detailed information about precision, recall and other metrics are available in the Additional file 2: Table S5

To assess the performance of the assemblers in capturing SNPs, we compared the F1-score calculated for the perfect assemblies (Fig. 7). This plot demonstrates the impact of downsampling on the resulting assemblies in terms of the F1 score, which combined precision and recall. Most tools performed best at a downsampling sequencing depth of 10X in terms of F1-score.

Among the assemblers, MitoFlex and GetOrganelle performed the best at a sequencing depth of 10X, consistently capturing the SNPs across all samples with an average F1-Score of 0.919. Additionally, MToolBox and NOVOPlasty performed consistently across all samples with an average F1-score of 0.897 and 0.890, respectively. Among these two, MToolBox consistently outperformed NOVOPlasty in capturing the SNPs present in the mitochondrial genome as calculated by the F1-score.

## Discussion

In this study, we evaluate the performance of ten short-read mitochondrial genome assemblers for assembling the human mitochondrial genome. The weightage of assessment criteria used in this study varies based on the downstream analysis. For instance, when the sample size is modest, the run time parameter might not be all that useful. Still, this becomes crucial when the ultimate objective is to assemble 100 or 1000 mitochondrial genomes. Thus, the primary evaluation criterion for a tool is whether it can generate quality assemblies; otherwise, if the tool is not functioning correctly, all other criteria are irrelevant. This study used the default parameters or parameters advised to be altered in the documentation. Recommendations regarding parameter usage made by developers and past users in the GitHub issues were also used, if required, after careful consideration and are otherwise stated. While it is possible that adjusting other advanced settings might lead to different results from these tools, the impact of such configuration was not explored in the current study.

MToolBox, NOVOPlasty and ORG.Asm are the only tools that produce high-quality assemblies for all the samples with varying sequencing depths. Based on F1-score MToolBox outperformed the other two tools with a mean F1-score of 0.897. Also, out of these three tools, only MToolBox supported additional features like variant calling, haplogroup detection etc., which can be utilized in many downstream analyses. Based on these characteristics MToolBox achieved the best performance overall, followed by NOVOPlasty. MitoFlex and GetOrganelle got the best performance overall in terms of F1-score, with a mean F1-score of 0.91 for the samples with a mean sequencing depth of 10X. So, it is advantageous to utilize MitoFlex or GetOrganelle when dealing with low-sequencing data.

### Guidelines for the end-user

From this study, we recommend that MToolBox may be used to reconstruct the mitochondrial genome from short-read whole genome sequencing data, especially if sufficient computing power is available and the sequencing depth varies. Use NOVOPlasty, the second-best tool, if MToolBox occasionally fails to produce the correct assembly. In the case of samples with low sequencing depth, MitoFlex and GetOrganelle can be utilized to get high-quality mitochondrial genome assemblies. Other options include manually adjusting the tool's parameters. Since NOVOplasty uses a single thread, running it in parallel will result in a shorter run time and a higher success rate for a large sample size.

### Ideas for future development

The statistics of average CPU utilization make it abundantly evident that not all of the tools in a mitochondrial assembly pipeline are making full use of the computational resources allocated by the user. Ergo, one possibility for future improvement is to alter the current tools to leverage the availability of computational resources. This will result in a shorter run time, increasing throughput given large datasets. Since most of the assemblers require other software to function, a decent strategy for future development and distribution would be to either containerize all the tools using docker, singularity, or conda package etc. or use workflow management languages such as Nextflow, Snakemake that allow reproducibility and reduce hassle for end users.

Finally, given that long-read sequencing is mainstream nowadays (Nanopore® 64 and PacBio® 65) and that the significant benefits of these technologies include repeat resolution 66 and identification of large structural variants 67, there is a need to develop tools that can take full advantage of long reads to generate better mitochondrial genomes for any species. Currently, only one reference-based assembler is available, Organelle_PBA, that effectively leverages the benefits offered by PacBio sequencing reads to assemble the organelle genomes. Furthermore, long-read sequencing has made remarkable progress, from generating high error rates to producing high-quality (Q30) reads 68. Therefore, moving forward, the most effective approach is to develop assemblers capable of harnessing high-quality long-read sequencing data to assemble the organelle genomes with resolved repeats for any given species.

## Conclusion

Whole genome sequencing data can be used to assemble mitochondrial genomes. The assembled genomes can find their application in tracing maternal ancestry, human migration and forensic analysis. Except for MToolBox, all the other tools explored in this study can be used to assemble the mitochondrial genomes from other species' whole genome sequencing data. Still, in this study, we only focused on assembling human mitochondrial genomes.

Nevertheless, this study demonstrates that not all techniques can assemble complete mitochondrial genomes effectively, irrespective of the kind of data (real or simulated) used as the input. The assemblies generated by MEANGS for simulated datasets exhibited fragmentation, resulting in relatively poor assembly scores. However, all the assemblies successfully captured the human mitochondrial genome's protein-coding genes (PCGs). The developers of MEANGS suggested that gaps in the assembly could be attributed to the low sequencing depth of the NGS data 39. However, in our study, the simulated datasets had mitochondrial sequencing depths ranging from 1000 to 4000X, yet MEANGS still struggled to produce complete assemblies like other tools. For WGS datasets of humans, Norgal and mitoMaker failed the initial testing of assembling the mitochondrial genomes. Norgal exceeded the time limit of 24 h for completion, and the resulting assembly had a length of approximately 73 Kbs. Similarly, mitoMaker didn't finish under the time limit when executed with 16 computational threads. Additional information regarding the failure of these two tools is provided in Additional file 3.

Given the varying success among these tools, our study highlighted the merits and drawbacks of each tool, enabling end users to make informed decisions. Furthermore, we have provided instructions to guide end users ineffectively utilizing these tools for their specific needs. This assessment was weighted on various parameters such as computational power, data size, run time and assembly quality. Our investigation has led us to the conclusion that it is now feasible to reconstruct thousands of mitochondrial genome assemblies using the available mitochondrial genome assemblers.

### Supplementary Information

**Additional file 1.** Details about the qualitative assessment of the human mitochondrial assemblers evaluated in this study.

**Additional file 2.** An Excel spreadsheet containing Tables S1–S6. **Table S1**. Computational metrics including run time, memory and CPU usage for simulated datasets. **Table S2**. Scores were calculated for the mitochondrial assemblies generated by all ten tools using the simulated dataset. **Table S3**. Information about sequencing depth and number of reads. **Table S4**. Scores were calculated for the mitochondrial assemblies based on publicly available datasets, including the down-sampled data. **Table S5**. Detailed information about True Positives, False Positives, Precision and other metrics. **Table S6**. Information about the Mitochondrial assemblers GitHub commits used in this benchmark study. **Table S7**. Comparison of perfect assemblies of all the sequencing depths for each assembler with the reference mitochondrial genome using QUAST.

**Additional file 3.** Details about the problems with Norgal and mitoMaker.

## Data Availability

The Supplementary materials, including program codes, simulated datasets, assemblies and other metrics, are available in the GitHub repository https://github.com/Nirmal2310/Mitochondrial_Benchmarking_study. The raw sequencing data used in this study is publicly available under the google cloud platform https://console.cloud.google.com/storage/browser/genomics-public-data/platinum-genomes/fastq?pageState=(%22StorageObjectListTable%22:(%22f%22:%22%255B%255D%22))&prefix=&forceOnObjectsSortingFiltering=false.

## References

[1] Stewart JB, Chinnery PF (2015). The dynamics of mitochondrial DNA heteroplasmy: implications for human health and disease. Nat Rev Genet.

[2] Anderson S (1981). Sequence and organization of the human mitochondrial genome. Nature.

[3] Lutz S, Weisser HJ, Heizmann J, Pollak S (1996). mtDNA as a tool for identification of human remains. Identification using mtDNA. Int. J. Legal Med..

[4] Calvo SE (2012). Molecular diagnosis of infantile mitochondrial disease with targeted next-generation sequencing. Sci Transl Med.

[5] Oota H, Saitou N, Matsushita T, Ueda S (1999). Molecular genetic analysis of remains of a 2,000-year-old human population in China-and its relevance for the origin of the modern Japanese population. Am J Hum Genet.

[6] Brown WM, Prager EM, Wang A, Wilson AC (1982). Mitochondrial DNA sequences of primates: tempo and mode of evolution. J Mol Evol.

[7] Andrews RM (1999). Reanalysis and revision of the Cambridge reference sequence for human mitochondrial DNA. Nat Genet.

[8] Green RE (2008). A complete Neandertal mitochondrial genome sequence determined by high-throughput sequencing. Cell.

[9] Behar DM (2012). A "Copernican" reassessment of the human mitochondrial DNA tree from its root. Am J Hum Genet.

[10] Bandelt H-J, Kloss-Brandstätter A, Richards MB, Yao Y-G, Logan I (2014). The case for the continuing use of the revised Cambridge Reference Sequence (rCRS) and the standardization of notation in human mitochondrial DNA studies. J Hum Genet.

[11] Chinnery PF, Hudson G (2013). Mitochondrial genetics. Br Med Bull.

[12] Stewart JB, Chinnery PF (2021). Extreme heterogeneity of human mitochondrial DNA from organelles to populations. Nat Rev Genet.

[13] Yao Y (2019). A simple method for sequencing the whole human mitochondrial genome directly from samples and its application to genetic testing. Sci Rep.

[14] Dhorne-Pollet S, Barrey E, Pollet N (2020). A new method for long-read sequencing of animal mitochondrial genomes: application to the identification of equine mitochondrial DNA variants. BMC Genom.

[15] Harrison RG (1989). Animal mitochondrial DNA as a genetic marker in population and evolutionary biology. Trends Ecol Evol.

[16] Keith Barker F (2014). Mitogenomic data resolve basal relationships among passeriform and passeridan birds. Mol Phylogenet Evol.

[17] Hebert PDN, Ratnasingham S, de Waard JR (2003). Barcoding animal life: cytochrome c oxidase subunit 1 divergences among closely related species. Proc Biol Sci.

[18] Kurabayashi A, Sumida M (2013). Afrobatrachian mitochondrial genomes: genome reorganization, gene rearrangement mechanisms, and evolutionary trends of duplicated and rearranged genes. BMC Genom.

[19] Smith DR (2016). The past, present and future of mitochondrial genomics: have we sequenced enough mtDNAs?. Brief Funct Genom.

[20] Wang T (2022). The Human Pangenome Project: a global resource to map genomic diversity. Nature.

[21] Yuan Y (2020). Comprehensive molecular characterization of mitochondrial genomes in human cancers. Nat Genet.

[22] Cann RL, Stoneking M, Wilson AC (1987). Mitochondrial DNA and human evolution. Nature.

[23] Alves-Silva J (2000). The ancestry of Brazilian mtDNA lineages. Am J Hum Genet.

[24] Denaro M (1981). Ethnic variation in Hpa 1 endonuclease cleavage patterns of human mitochondrial DNA. Proc Natl Acad Sci USA.

[25] Taylor RW, Turnbull DM (2005). Mitochondrial DNA mutations in human disease. Nat Rev Genet.

[26] Budowle B, Allard MW, Wilson MR, Chakraborty R (2003). Forensics and mitochondrial DNA: applications, debates, and foundations. Annu Rev Genomics Hum Genet.

[27] Bruce MW, Allard MR, Wilson R (2003). Forensics and mitochondrial DNA: applications, debates, and foundations. Annu Rev Genom Hum Genet.

[28] Lischer HEL, Shimizu KK (2017). Reference-guided de novo assembly approach improves genome reconstruction for related species. BMC Bioinform.

[29] Calabrese C (2014). MToolBox: a highly automated pipeline for heteroplasmy annotation and prioritization analysis of human mitochondrial variants in high-throughput sequencing. Bioinformatics.

[30] Al-Nakeeb K, Petersen TN, Sicheritz-Pontén T (2017). Norgal: extraction and de novo assembly of mitochondrial DNA from whole-genome sequencing data. BMC Bioinform.

[31] Meng G, Li Y, Yang C, Liu S (2019). MitoZ: a toolkit for animal mitochondrial genome assembly, annotation and visualization. Nucleic Acids Res.

[32] Lopez JV, Yuhki N, Masuda R, Modi W, O'Brien SJ (1994). Numt, a recent transfer and tandem amplification of mitochondrial DNA to the nuclear genome of the domestic cat. J Mol Evol.

[33] Dierckxsens N, Mardulyn P, Smits G (2017). NOVOPlasty: de novo assembly of organelle genomes from whole genome data. Nucleic Acids Res.

[34] Lee YS, Kim W-Y, Ji M, Kim JH, Bhak J (2009). MitoVariome: a variome database of human mitochondrial DNA. BMC Genom.

[35] Docker. https://hub.docker.com/repositories/nirmal2310.

[36] Weber LM (2019). Essential guidelines for computational method benchmarking. Genome Biol.

[37] *ARC: Assembly by Reduced Complexity (ARC)*. (Github).

[38] Jin J-J (2020). GetOrganelle: a fast and versatile toolkit for accurate de novo assembly of organelle genomes. Genome Biol.

[39] Song M-H, Yan C, Li J-T (2022). MEANGS: an efficient seed-free tool for de novo assembling animal mitochondrial genome using whole genome NGS data. Brief Bioinform.

[40] Bakker FT (2015). Herbarium genomics: plastome sequence assembly from a range of herbarium specimens using an Iterative Organelle Genome Assembly pipeline. Biol J Linn Soc Lond.

[41] Hahn C, Bachmann L, Chevreux B (2013). Reconstructing mitochondrial genomes directly from genomic next-generation sequencing reads—a baiting and iterative mapping approach. Nucleic Acids Res.

[42] Li J-Y, Li W-X, Wang A-T, Yu Z (2021). MitoFlex: an efficient, high-performance toolkit for animal mitogenome assembly, annotation, and visualization. Bioinformatics.

[43] ORG.Asm / ORG.Asm. *GitLab* https://git.metabarcoding.org/org-asm/org-asm.

[44] Freudenthal JA (2020). A systematic comparison of chloroplast genome assembly tools. Genome Biol.

[45] Yu R (2023). De novo assembly and comparative analyses of mitochondrial genomes in Piperales. Genome Biol. Evol..

[46] Milián-García Y (2022). Mitochondrial genome sequencing, mapping, and assembly benchmarking for Culicoides species (Diptera: Ceratopogonidae). BMC Genom.

[47] Soorni A, Haak D, Zaitlin D, Bombarely A (2017). Organelle_PBA, a pipeline for assembling chloroplast and mitochondrial genomes from PacBio DNA sequencing data. BMC Genom.

[48] Schomaker-Bastos A, Prosdocimi F. mitoMaker: a pipeline for automatic assembly and annotation of animal mitochondria using raw NGS data. (2018) 10.20944/preprints201808.0423.v1

[49] Alqahtani F, Măndoiu II (2020). Statistical mitogenome assembly with RepeaTs. J Comput Biol.

[50] Conda — conda documentation. https://docs.conda.io/en/latest/.

[51] Nurk S (2022). The complete sequence of a human genome. Science.

[52] Gourlé H, Karlsson-Lindsjö O, Hayer J, Bongcam-Rudloff E (2019). Simulating Illumina metagenomic data with InSilicoSeq. Bioinform.

[53] Watson E, Davis R, Sue CM (2020). New diagnostic pathways for mitochondrial disease. J Transl Genet Genom.

[54] BioProject. https://www.ncbi.nlm.nih.gov/bioproject/PRJEB3381.

[55] Review criteria — JOSS documentation. https://joss.readthedocs.io/en/latest/review_criteria.html.

[56] Li H (2018). Minimap2: pairwise alignment for nucleotide sequences. Bioinformatics.

[57] Quinlan AR, Hall IM (2010). BEDTools: a flexible suite of utilities for comparing genomic features. Bioinformatics.

[58] BBMap. *SourceForge* https://sourceforge.net/projects/bbmap/ (2022).

[59] Gurevich A, Saveliev V, Vyahhi N, Tesler G (2013). QUAST: quality assessment tool for genome assemblies. Bioinformatics.

[60] Weissensteiner H (2016). mtDNA-Server: next-generation sequencing data analysis of human mitochondrial DNA in the cloud. Nucleic Acids Res..

[61] Ip EKK (2022). Benchmarking the effectiveness and accuracy of multiple mitochondrial DNA variant callers: practical implications for clinical application. Front Genet.

[62] Marçais G (2018). MUMmer4: a fast and versatile genome alignment system. PLoS Comput Biol.

[63] GitHub - MatteoSchiavinato/all2vcf: Toolkit to convert the output of common variant calling programs to VCF. *GitHub* https://github.com/MatteoSchiavinato/all2vcf

[64] Oxford Nanopore technologies. *Oxford Nanopore Technologies* https://nanoporetech.com/

[65] PacBio - sequence with confidence. *PacBio* https://www.pacb.com/ (2015)

[66] Amarasinghe SL (2020). Opportunities and challenges in long-read sequencing data analysis. Genome Biol.

[67] Begum G (2021). Long-read sequencing improves the detection of structural variations impacting complex non-coding elements of the genome. Int J Mol Sci.

[68] Kovaka S, Ou S, Jenike KM, Schatz MC (2023). Approaching complete genomes, transcriptomes and epi-omes with accurate long-read sequencing. Nat Methods.

