# Supplementary material for: A systematic comparison of human mitochondrial genome assembly tools
Source: BMC Bioinformatics. 2023 Sep 13;24:341. doi: 10.1186/s12859-023-05445-3 (PMC10498642; doi:10.1186/s12859-023-05445-3)
Supplement: Supplementary file 1 — Additional file 1. Details about the qualitative assessment of the human mitochondrial assemblers evaluated in this study. [file 12859_2023_5445_MOESM1_ESM.docx]

**Details about the output obtained for Norgal and mitoMaker**

1. **
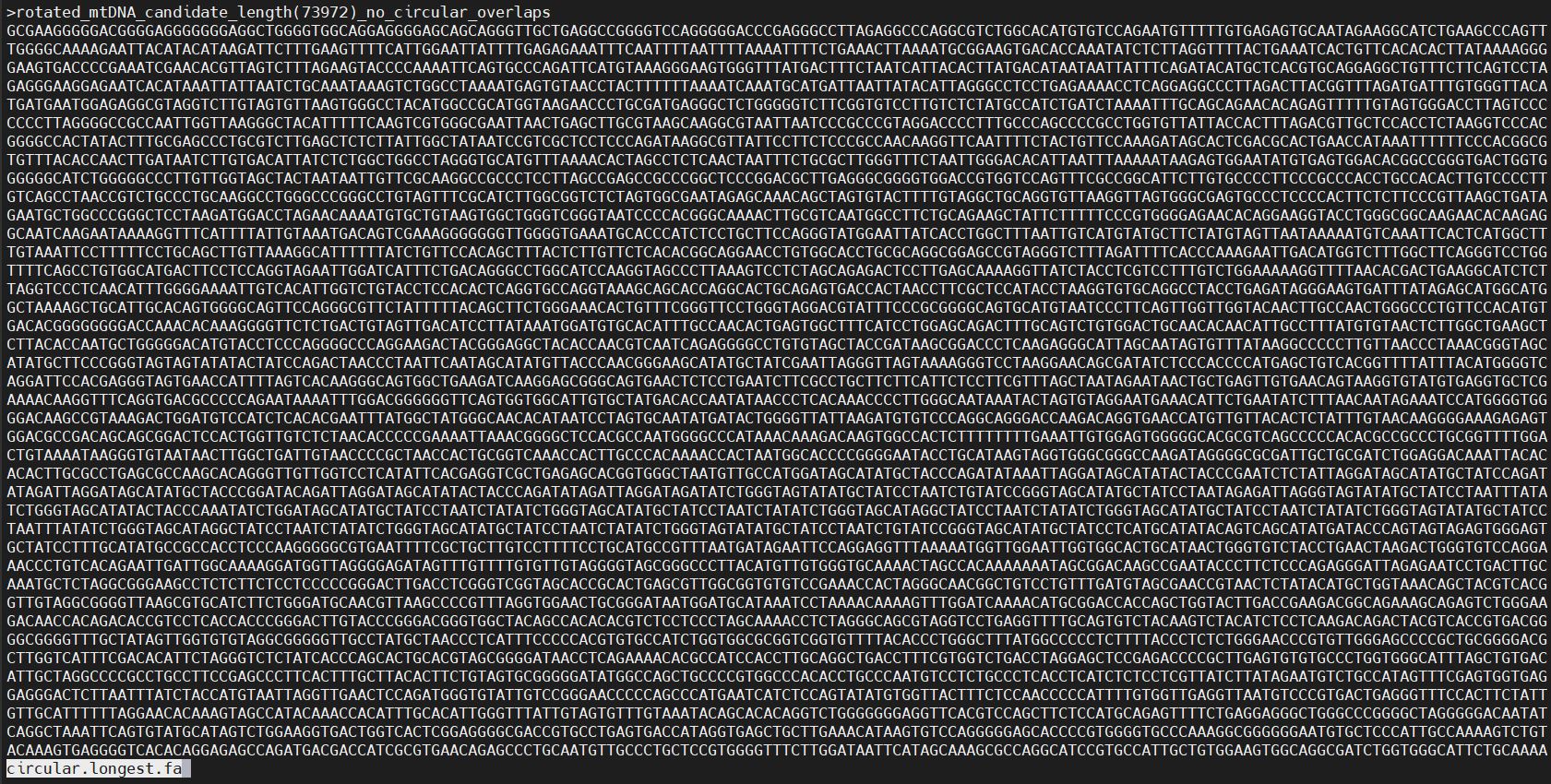
Norgal:**

Resulting Assembly (73,972 bases) obtained from Norgal assembler with Whole Genome Dataset NA12877 from the study “Whole genome sequencing and variant calls from Coriell CEPH/UTAH 1463 family to create a platinum standard comprehensive set for variant calling improvement^1^.”

The obtained assembly was mapped to the Nucleotide Collection database using web-based Nucleotide BLAST.^2^


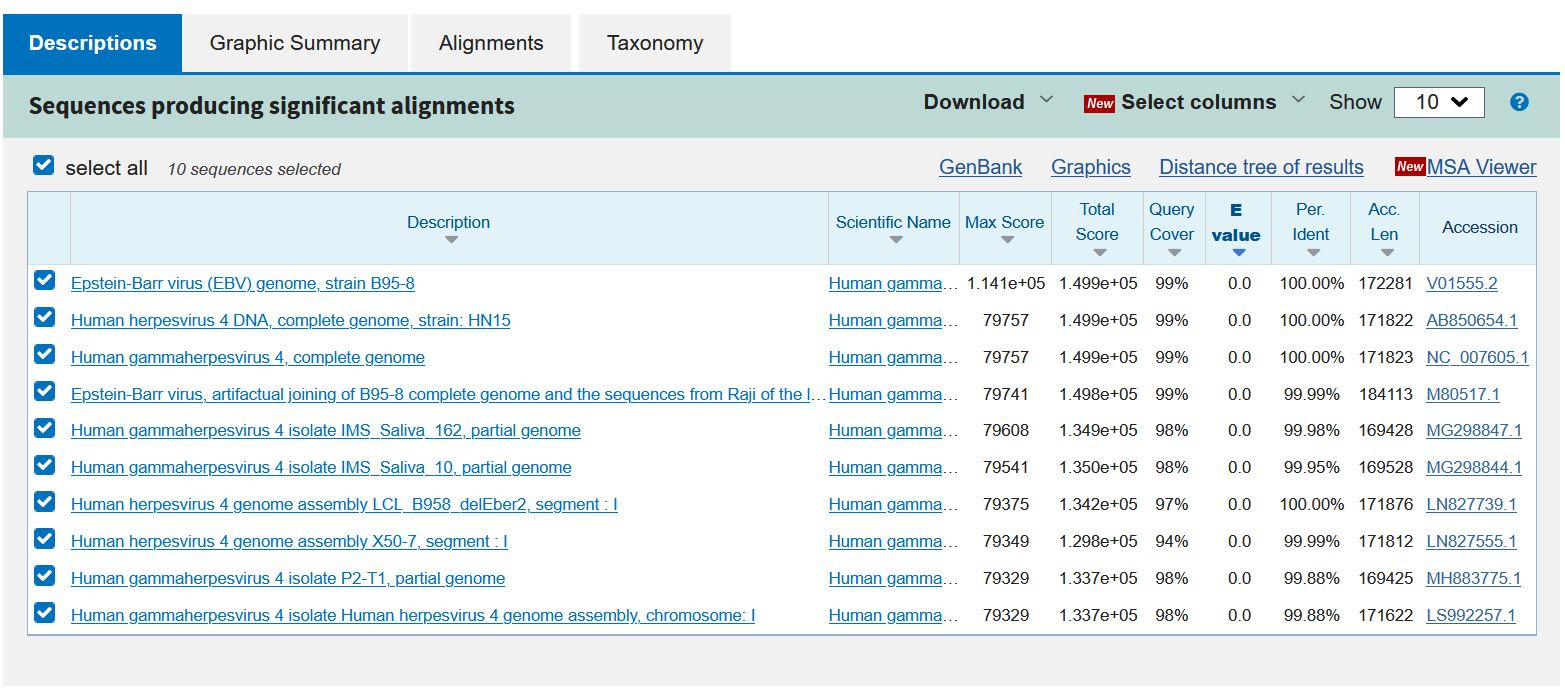


The resulting assembly was not mapping to the human mitochondrial genome.

1. **mitoMaker:**

**
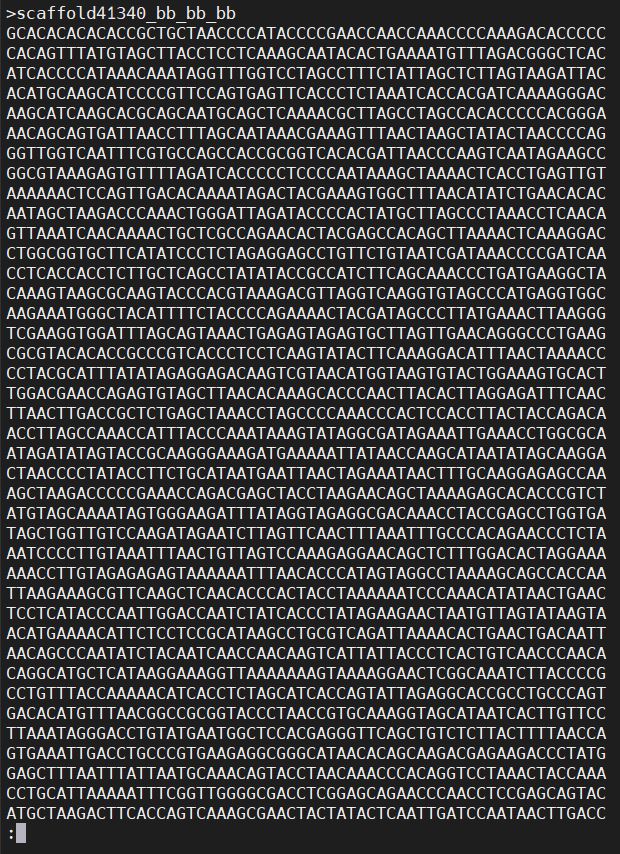
**

Resulting assembly (16748 bases) obtained from Mitomaker assembler with Whole Genome Sequencing Dataset NA12877 from the study “Whole genome sequencing and variant calls from Coriell CEPH/UTAH 1463 family to create a platinum standard comprehensive set for variant calling improvement^1^.”

The obtained assembly was then mapped to the Nucleotide database using web-based Nucleotide BLAST.^2^


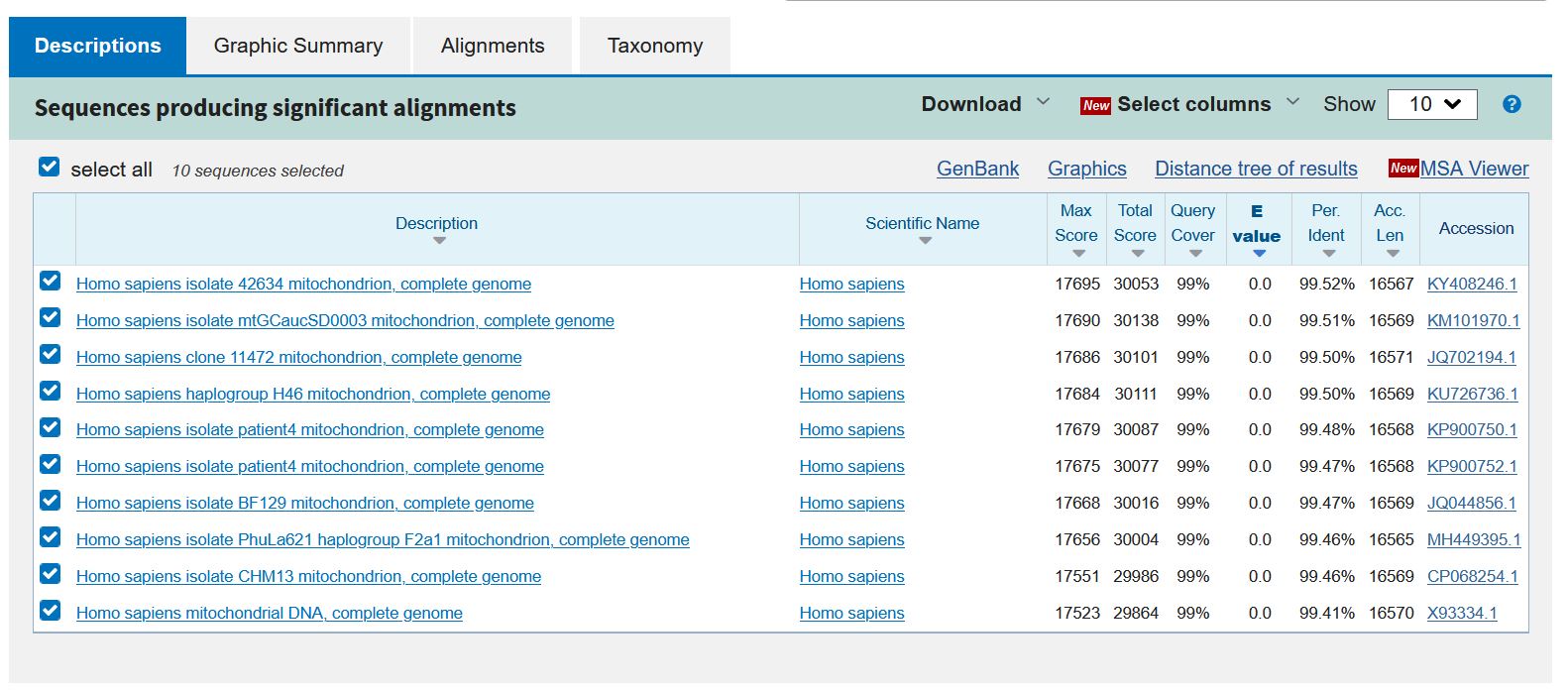


The resulting assembly was mapping to the human mitochondrial genomes.

| Tool | Computation Threads Used | Execution Time | ASSEMBLY  MODE | Longest Contig (bp) |
| --- | --- | --- | --- | --- |
| Norgal | 16 | 81:28:39 | *de novo*, K-mer based | 73972 |
| MitoMaker | 16 | 44:58:9 | *de novo*, K-mer based | 16748 |

**Table 1:** Time taken by the Norgal and MitoMaker. Both the tools exceeded the time limit of 24 hours.

1. BioProject. https://www.ncbi.nlm.nih.gov/bioproject/PRJEB3381.

2. Nucleotide BLAST: Search nucleotide databases using a nucleotide query. https://blast.ncbi.nlm.nih.gov/Blast.cgi?PROGRAM=blastn&BLAST_SPEC=GeoBlast&PAGE_TYPE=BlastSearch.
