# Supplementary material for: A systematic comparison of human mitochondrial genome assembly tools
Source: BMC Bioinformatics. 2023 Sep 13;24:341. doi: 10.1186/s12859-023-05445-3 (PMC10498642; doi:10.1186/s12859-023-05445-3)
Supplement: Supplementary file 3 — Additional file 3. Details about the problems with Norgal and mitoMaker. [file 12859_2023_5445_MOESM3_ESM.docx]

### Qualitative Assessment of Ten Short Read-Based Mitochondrial Assemblers

The qualitative assessment of all the tools used in this study was based on the reviewer guidelines for the Journal of Open-Source Software (JOSS)^1^. The evaluation was done based on the following questions:

1. Are the tools easy to install?

2. Is there proper documentation for running the tool or a test dataset to check the installation?

3. Is the tool well maintained (issues answered, continuous update)?

4. Is the tool Open Source?

All these questions were answered "Good", "Okay", and "Bad" based on various aspects of the tools that are being described here.

1. Installing the required tools is crucial while setting up a computational pipeline. In the first question, we have judged the tools based on this functionality. The tool was categorized as " Good " if installed using any package manager like Anaconda, pip or apt, packaged into a container like Docker, Singularity, Snakemake or Nextflow, or has precompiled binaries available. The tool was categorized as "Okay" if the installation script was given with the tool and all the dependencies were installed using a single script. However, if the installation was unsuccessful in one go, had dependencies conflict and required multiple debugging steps, then the tools were annotated as bad.
2. Usually, multiple parameters are available for a single tool, and testing each parameter will require a lot of time and effort. So, it is essential to provide the documentation of the tool, which will serve as the starting point to get started. Also, the test datasets will confirm the successful installation and execution of the tool and provide the parameters to be used while running the tool for a new dataset. All the tools considered in this study were also judged based on these two factors. The tool was categorized as "Good" if the documentation was available. Every parameter of the tool is explained in the documentation, and the test cases for running the tool are also available in the tool's repository. It was categorized as "Okay" if the documentation was available with all the explanations. Still, the test datasets are not given; links to download one example and the command to run the analysis are given in the documentation. The tool was categorized as "Bad" if the documentation did not comprehensively explain the parameters and lacked the test dataset to check the installation status.
3. The field of computational biology is dynamic, with new technologies, file formats, etcetera. Any tool needs to be up to date with the latest features and the ability to handle a variety of datasets. For this, it is required that the tool is regularly maintained and updated, and we assess the tools for this feature as well. The tool was annotated "Good" if the issues raised in its GitHub repository are being answered regularly and updated regularly to fix the bugs found by the users. The tool was annotated "Okay" if it is not getting updated, but the issues are continuously getting resolved in the public repository. Lastly, the tool was categorized as "Bad" if it was neither getting regular updates nor resolving the errors raised.
4. All the tools assessed in this study are open-source. The main advantage of open-source tool development is that it promotes transparency, supports collaboration, and all the bugs and issues can be raised by the users, which helps improve the tool.

1. Review criteria — JOSS documentation. https://joss.readthedocs.io/en/latest/review_criteria.html.
